# The prognostic utility of prehospital qSOFA in addition to emergency department qSOFA for sepsis in patients with suspected infection: A retrospective cohort study

**DOI:** 10.1371/journal.pone.0282148

**Published:** 2023-02-24

**Authors:** Ayaka Saito, Itsuki Osawa, Junichiro Shibata, Tomohiro Sonoo, Kensuke Nakamura, Tadahiro Goto

**Affiliations:** 1 Faculty of Medicine, The University of Tokyo, Tokyo, Japan; 2 Department of Emergency and Critical Care Medicine, The University of Tokyo Hospital, Tokyo, Japan; 3 TXP Medical Co. Ltd., Tokyo, Japan; 4 Department of Emergency and Critical Care Medicine, Hitachi General Hospital, Ibaraki, Japan; 5 Department of Clinical Epidemiology and Health Economics, School of Public Health, The University of Tokyo, Tokyo, Japan; Universitair Kinderziekenhuis Koningin Fabiola: Hopital Universitaire des Enfants Reine Fabiola, BELGIUM

## Abstract

**Background:**

The quick sequential organ failure assessment (qSOFA) was widely used to estimate the risks of sepsis in patients with suspected infection in the prehospital and emergency department (ED) settings. Due to the insufficient sensitivity of qSOFA on arrival at the ED (ED qSOFA), the Surviving Sepsis Campaign 2021 recommended against using qSOFA as a single screening tool for sepsis. However, it remains unclear whether the combined use of prehospital and ED qSOFA improves its sensitivity for identifying patients at a higher risk of sepsis at the ED.

**Methods:**

We retrospectively analyzed the data from the ED of a tertiary medical center in Japan from April 2018 through March 2021. Among all adult patients (aged ≥18 years) transported by ambulance to the ED with suspected infection, we identified patients who were subsequently diagnosed with sepsis based on the Sepsis-3 criteria. We compared the predictive abilities of prehospital qSOFA, ED qSOFA, and the sum of prehospital and ED qSOFA (combined qSOFA) for sepsis in patients with suspected infection at the ED.

**Results:**

Among 2,407 patients with suspected infection transported to the ED by ambulance, 369 (15%) patients were subsequently diagnosed with sepsis, and 217 (9%) died during hospitalization. The sensitivity of prehospital qSOFA ≥2 and ED qSOFA ≥2 were comparable (c-statistics for sepsis [95%CI], 0.57 [0.52–0.62] vs. 0.55 [0.50–0.60]). However, combined qSOFA (cutoff, ≥3 [max 6]) was more sensitive than ED qSOFA (cutoff, ≥2) for identifying sepsis (0.67 [95%CI, 0.62–0.72] vs. 0.55 [95%CI, 0.50–0.60]). Using combined qSOFA, we identified 44 (12%) out of 369 patients who were subsequently diagnosed with sepsis, which would have been missed using ED qSOFA alone.

**Conclusions:**

Using both prehospital and ED qSOFA could improve the screening ability of sepsis among patients with suspected infection at the ED.

## Introduction

Early identification of suspected sepsis and initiation of appropriate management play a crucial role in reducing the mortality of sepsis [1, 2]. To screen patients at high risk of sepsis among patients with suspected infection at the emergency department (ED), the Sepsis-3 Task Force recommended the use of the quick Sequential Organ Failure Assessment (qSOFA) score (i.e., qSOFA ≥2 should be considered as suspected sepsis) [3], a simple algorithm that has been widely used in the ED setting. Moreover, as qSOFA was originally developed for use outside the intensive care unit (ICU), discussions have been made to improve the quality of triage by using qSOFA in the prehospital setting [4–7].

Despite the initial recommendation to use qSOFA by the Sepsis-3 Task Force, several studies have shown that qSOFA is more specific but less sensitive compared with other screening tools (e.g., the systemic inflammatory response syndrome [SIRS] criteria) in predicting prognostic outcomes related to sepsis [8–12]. Thus, the Surviving Sepsis Campaign 2021 recommended against using qSOFA as a single screening tool for sepsis or septic shock [13].

The advantage of qSOFA is its simplicity compared to other scoring systems for estimating the risk of sepsis (e.g., SIRS criteria, National Early Warning Score [NEWS]). Thus, it would be valuable to devise a new scoring system that improves on qSOFA with its simplicity preserved. As Keivlan et al. reported that repeated measurements of qSOFA improved its predictive validity for sepsis compared to a single measurement of qSOFA [14], it may be beneficial to consider the combined use of prehospital and ED qSOFA for accurately screening patients at a higher risk of sepsis on arrival at the ED. Nonetheless, it remains unclear whether the combined use of prehospital and ED qSOFA improves its sensitivity for identifying patients at a higher risk of sepsis at the ED.

To address this knowledge gap in the literature, we aimed to clarify whether the addition of prehospital qSOFA to ED qSOFA improves the predictive ability for sepsis in patients with suspected infection at the ED compared with the use of prehospital qSOFA or ED qSOFA alone.

## Methods

### Study design and setting

This is a retrospective cohort study using data from the ED of Hitachi General Hospital from April 1, 2018, to March 31, 2021. Hitachi General Hospital is a tertiary medical center in Japan that covers a population of approximately 3 million people and has approximately 20,000 ED visits annually. The medical records are structured through an electronic medical information system (the NEXT Stage ER system, TXP Medical Co. Ltd., Tokyo, Japan), which supports healthcare professionals including ambulance officers entering clinical information as structured data [15]. The study protocol was approved by the ethics committee of Hitachi General Hospital, which waived the requirement for informed consent due to the retrospective nature of the study.

### Study participants

**Fig 1:**
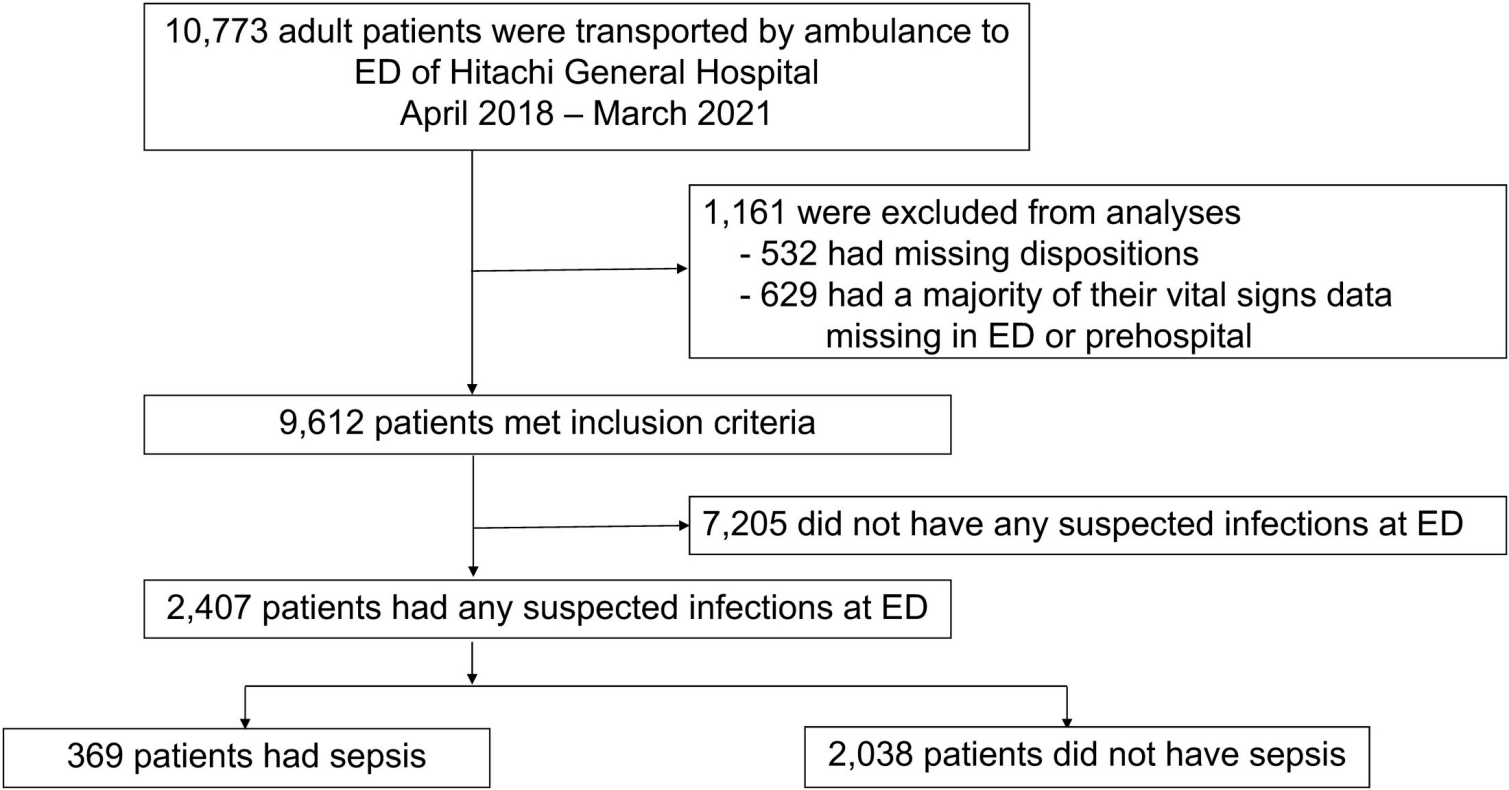
Flow diagram of study participants selection for analysis. Among 10,773 patients who were transported by ambulance to the ED of Hitachi General Hospital from April 2018 to March 2021, we identified 2,407 patients with suspected infection whose prehospital and ED vital signs data were available. Abbreviations: ED, Emergency Department.

We identified adult patients (aged ≥18 years) with suspected infection transported to the ED by ambulance and initially treated by emergency medicine physicians. We defined suspected infection based on the chief complaint of fever, high body temperature (≥37.5°C), or ED diagnosis of infection (e.g., pneumonia, urinary tract infection, cellulitis, pharyngitis, and meningitis). The diagnosis of infection was inferred from each patient’s clinical context on arrival at the ED, which was routinely recorded by emergency physicians at Hitachi General Hospital and automatically converted to the International Classification of Diseases (ICD) -10 codes by the algorithm of the NEXT Stage ER system [15]. We excluded the following patients from the analysis: patients who had trauma or cardiac arrest [16], died immediately upon arrival at the ED, were transported to other hospitals after the ED arrival, and had six or more missing parameters out of seven vital sign parameters (systolic blood pressure [sBP], diastolic blood pressure [dBP], heart rate [HR], respiratory rate [RR], body temperature [BT], altered mental status [Glasgow coma scale (GCS) ≤14], and the O2 saturation level [SpO2]) either at prehospital or at ED arrival (**Fig 1**).

### Measurements

For each enrolled patient, we collected patient age, sex, vital signs both in the prehospital and ED settings (sBP, dBP, HR, RR, BT, altered mental status [GCS ≤14], and SpO_2_), receipt of oxygen therapy upon arrival at the ED, and disposition (discharge from the ED, ICU admission, and in-hospital mortality). For patients admitted to the ICU, all data on laboratory tests (e.g., serum creatinine, total bilirubin, platelet count, and serum lactate level), microbiological blood culture tests, and the use of medications (e.g., antibiotics and vasopressors), and mechanical ventilators were also collected to determine the presence of sepsis based on the sepsis clinical surveillance definition (**S1 Appendix**) [17].

### Outcome measures

The primary outcome was the diagnosis of sepsis based on the Sepsis-3 criteria [3]. We identified patients with sepsis using the modified sepsis clinical surveillance definition (shown in **S1 Appendix**) instead of using the original Sepsis-3 criteria, since we had difficulty accurately identifying patients with sepsis based on the Sepsis-3 criteria (i.e., ≥2-point increase in SOFA score) in the current study due to its retrospective nature. We utilized the definition of sepsis based on the criteria initially proposed by Rhee et al. [17], which was to define sepsis based on the Sepsis-3 criteria in retrospective studies [18, 19]. At Hitachi General Hospital, as a general practice, patients who visit the ED that need to be hospitalized are first treated by intensivists in the ICU, and then stepped down depending on their medical condition. In other words, all patients with suspected infection at the ED are always admitted to the ICU (i.e., not initially admitted to the general wards) for treatment in our hospital system. Therefore, in this study, we identified patients subsequently diagnosed with sepsis admitted to the ICU, which means all patients with sepsis hospitalized via the ED [19, 20].

### Statistical analyses

We computed summary statistics to delineate the characteristics of all patients with suspected infection. All the data we used for the calculation of qSOFA, including the number of missing data, are shown in **Table 1**. In order to minimize the selection bias induced by the missing data, the random forest method was applied to impute all the missing vital signs data using the following variables: age, sex, vital signs both at prehospital and ED arrival (sBP, dBP, HR, RR, BT, GCS, and SpO_2_), receipt of oxygen therapy upon arrival at the ED, and outcomes (i.e., sepsis, septic shock, and in-hospital mortality) [21–23]. After the imputation of all the missing vital signs data, we calculated both the prehospital and ED qSOFA.

**Table 1:** The proportion of missing variables in 2,407 patients.

Variables	Missing, n (%)*
**Vital signs at ED arrival**	
Systolic blood pressure	108 (4)
Diastolic blood pressure	114 (5)
Heart rate	113 (5)
Respiratory rate	184 (8)
Body temperature	102 (4)
Altered mental status [GCS ≤14]	787 (33)
**Prehospital vital signs**	
Systolic blood pressure	186 (8)
Diastolic blood pressure	224 (9)
Heart rate	47 (2)
Respiratory rate	262 (11)
Body temperature	181 (8)
Altered mental status [GCS ≤14]	56 (2)

Abbreviations: ED, emergency department; GCS, Glasgow Coma Scale; qSOFA, The quick Sequential Organ Failure Assessment

*All missing data were imputed with the random forest method. In our analyses, we imputed all qSOFA using all vital signs data with multiple imputation estimations by the random forest method. Altered mental status was defined as the GCS ≤14.

To assess the predictive abilities for sepsis of prehospital qSOFA, ED qSOFA, and the sum of prehospital and ED qSOFA (“combined qSOFA”), we first calculated c-statistics (i.e., the area under the receiver operating characteristics [ROC] curve) and prospective prediction results (i.e., sensitivity, specificity, positive predictive value [PPV], and negative predictive value [NPV]) for each threshold (e.g., combined qSOFA of ≥1, 2, 3, 4, 5, and 6). Next, we compared the predictive abilities of prehospital qSOFA, ED qSOFA, and combined qSOFA. DeLong’s test was used to compare each ROC curve [24].

To verify our findings, we performed a sensitivity analysis using septic shock and in-hospital mortality as prognostic outcomes related to sepsis. We calculated c-statistics and prospective prediction results and compared the predictive abilities of prehospital qSOFA, ED qSOFA, and combined qSOFA for septic shock and in-hospital mortality, all in the same manner as the primary outcome.

All analyses were conducted using R version 4.1.0 (R Foundation, Vienna, Austria) [25]. A P-value less than 0.05 was considered statistically significant.

## Results

### Patient characteristics

Among 10,773 patients who were transported by ambulance to the ED of Hitachi General Hospital from April 2018 to March 2021, we identified 2,407 patients with suspected infection whose prehospital and ED vital signs data were available. The flow chart of the study participants’ selection process is shown in **Fig 1**. The median age (IQR) was 78 (67–85) years, and 1,393 (58%) were male. Of these, we identified 369 (15%) patients who developed sepsis, 133 (6%) patients who developed septic shock, and 217 (9%) patients who died during hospitalization. (**Table 2**).

**Table 2:** Characteristics of 2,407 patients with suspected infection.

Characteristics	All patients with suspected infection (n = 2407)
Age (year), median (IQR)	78 (67–85)
Male gender, No. (%)	1,393 (58)
**Vital signs at ED arrival**	
Systolic blood pressure (mmHg), median (IQR)	138 (117–159)
Diastolic blood pressure (mmHg), median (IQR)	80 (69–93)
Heart rate (/min), median (IQR)	97 (84–112)
Respiratory rate (/min), median (IQR)	22 (19–26)
Body temperature (°C), median (IQR)	37.7 (37.0–38.5)
Altered mental status [GCS ≤14], No. (%)	1092 (45)
**Prehospital vital signs**	
Systolic blood pressure (mmHg), median (IQR)	138 (118–160)
Diastolic blood pressure (mmHg), median (IQR)	78 (65–93)
Heart rate (/min), median (IQR)	100 (85–115)
Respiratory rate (/min), median (IQR)	24 (20–26)
Body temperature (°C), median (IQR)	37.5 (36.8–38.5)
Altered mental status [GCS ≤14], No. (%)	1129 (47)
**Prehospital qSOFA score,** No. (%)	
0	515 (21)
1	996 (41)
2	746 (31)
3	150 (6)
**Outcomes**	
Sepsis, No. (%)	369 (15)
Septic shock, No. (%)	133 (6)
In-hospital mortality, No. (%)	217 (9)

Abbreviations: IQR, interquartile range; ED, Emergency Department; GCS, Glasgow Coma Scale; qSOFA, The quick Sequential Organ Failure Assessment

### Main results

**Fig 2:**
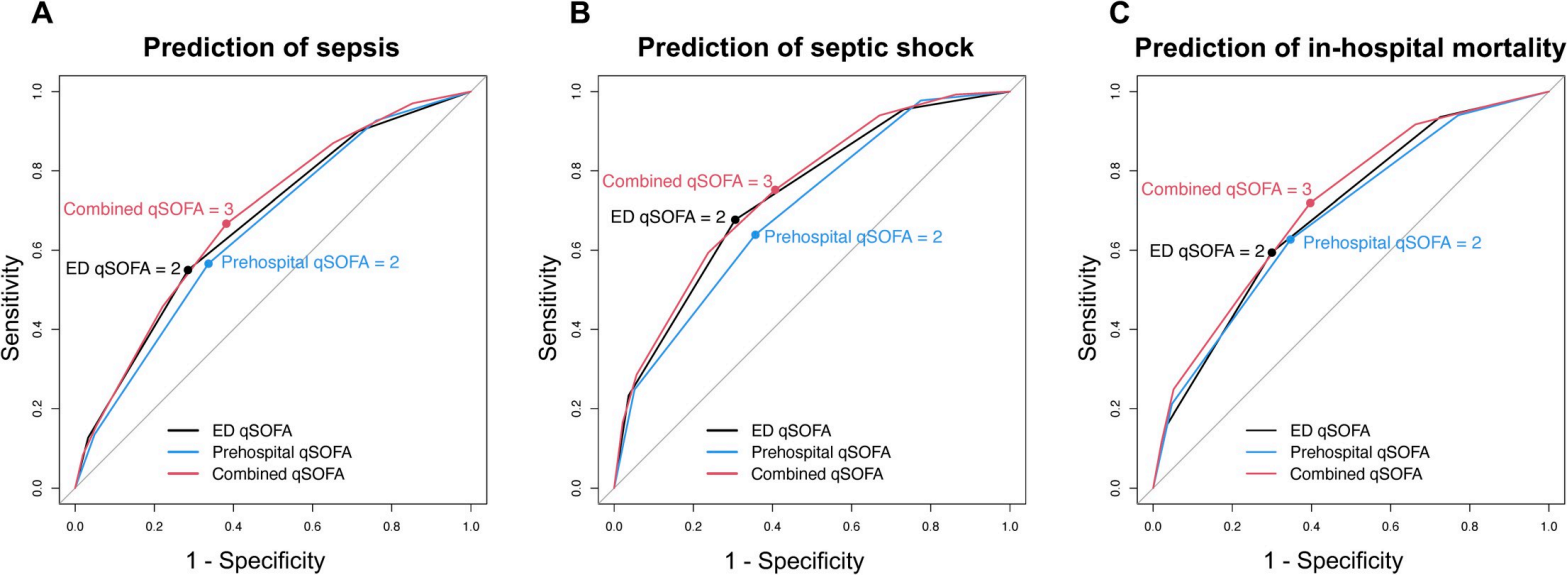
Receiver-operating-characteristics (ROC) curves of qSOFA among patients with clinically suspected infection. **(A)** Prediction of sepsis **(B)** Prediction of septic shock **(C)** Prediction of in-hospital mortality. The corresponding values of the area under the receiver operating characteristics curve for each model (i.e., the c-statistics) are presented in Tables 3–5. Abbreviations: ED, Emergency Department; qSOFA, The quick Sequential Organ Failure Assessment.

**Fig 3:**
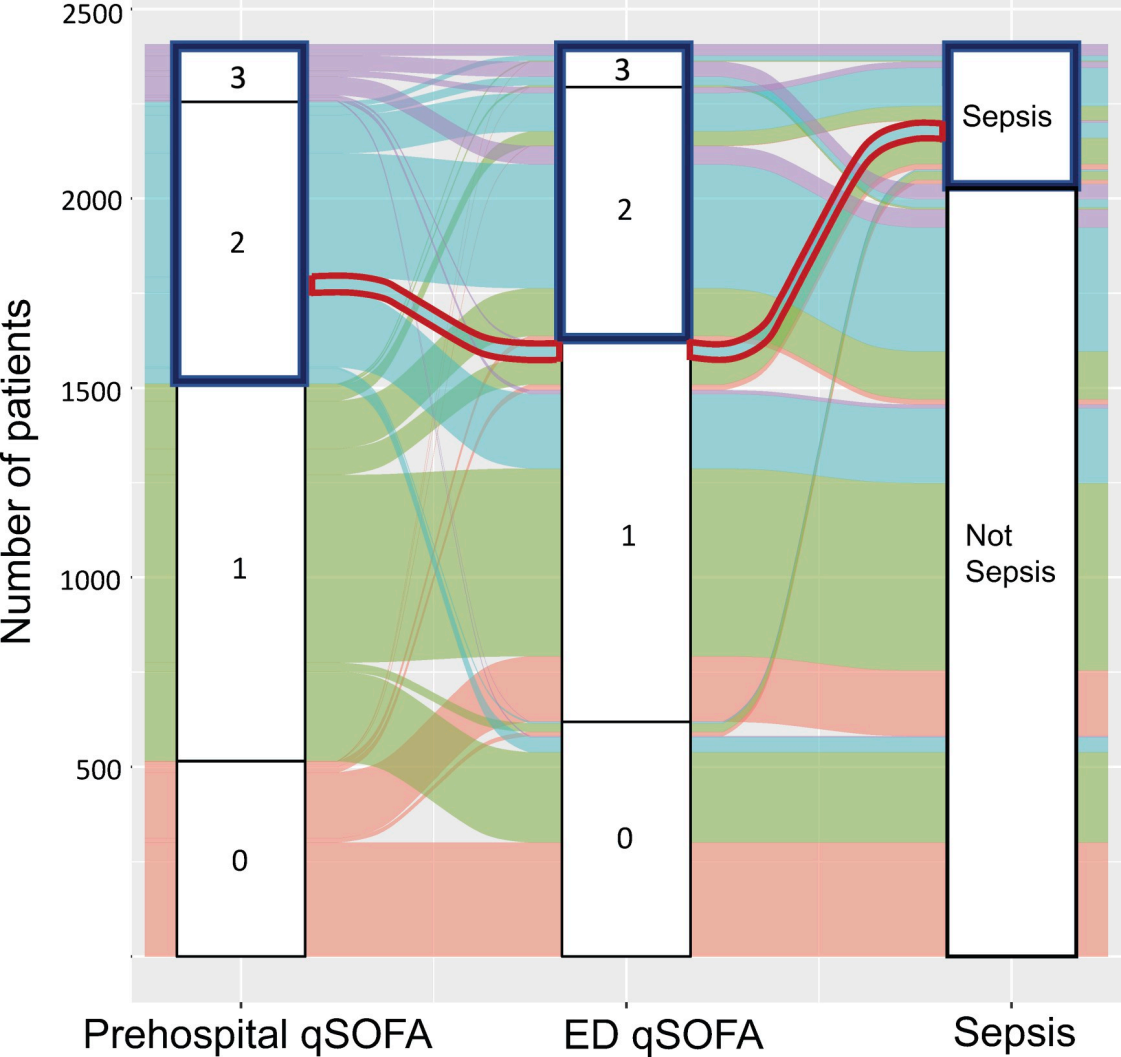
Alluvial plot of changes in prehospital and ED qSOFA scores and the diagnosis of sepsis among patients with clinically suspected infection. This alluvial plot illustrates the changes in qSOFA scores from prehospital to ED arrival and the diagnoses of sepsis based on the Sepsis-3 criteria among patients with clinically suspected infection. As represented by the area in red, combined qSOFA was able to identify 44 (12%) out of 369 patients who were subsequently diagnosed with sepsis based on the Sepsis-3 criteria, which would have been missed using ED qSOFA alone. Abbreviations: ED, Emergency Department; qSOFA, The quick Sequential Organ Failure Assessment.

The predictive ability of prehospital qSOFA for sepsis was almost equivalent to that of ED qSOFA (c-statistics of prehospital qSOFA versus ED qSOFA for sepsis [95%CI], 0.65 [0.62–0.68] vs. 0.67 [0.64–0.70], P-value 0.15; **Table 3 and Fig 2**), and the sensitivity of prehospital qSOFA ≥2 and ED qSOFA ≥2 were also comparable (sensitivity of prehospital qSOFA versus ED qSOFA for sepsis [95%CI], 0.57 [0.52–0.62] vs. 0.55 [0.50–0.60]; **Table 3**). The predictive ability of combined qSOFA was similar to that of ED qSOFA for identifying sepsis (c-statistics of combined qSOFA versus ED qSOFA for sepsis [95%CI], 0.68 [0.66–0.71] vs. 0.67 [0.64–0.70], P-value 0.03; **Table 3 and Fig 2**), whereas combined qSOFA (cutoff, ≥3) was significantly more sensitive than ED qSOFA (cutoff, ≥2) (sensitivity of combined qSOFA versus ED qSOFA for sepsis [95%CI], 0.67 [0.62–0.72] vs. 0.55 [0.50–0.60]; **Table 3**). **Fig 3** is an alluvial plot illustrating the changes in qSOFA scores from prehospital to ED arrival and the diagnoses of sepsis based on the Sepsis-3 criteria among all patients with clinically suspected infection. The area in red in **Fig 3** represents a group of patients who had qSOFA of 2 in the prehospital setting but 1 in the ED setting, and were subsequently diagnosed with sepsis. Therefore, by using combined qSOFA, we were able to identify 44 (12%) out of 369 patients who were subsequently diagnosed with sepsis who would otherwise have been missed using ED qSOFA alone.

**Table 3:** Predictive abilities of the ED qSOFA, prehospital qSOFA, and combined qSOFA for sepsis.

	Cutoff	c-statistics	P-value*	Sensitivity	Specificity	PPV	NPV
**ED qSOFA (95% CI)**	≥ 1	0.67 (0.64–0.70)	-	0.90 (0.87–0.93)	0.28 (0.27–0.30)	0.19 (0.17–0.20)	0.94 (0.92–0.96)
	≥ 2			0.55 (0.50–0.60)	0.71 (0.70–0.73)	0.26 (0.23–0.29)	0.90 (0.88–0.91)
	≥ 3			0.13 (0.09–0.16)	0.97 (0.96–0.98)	0.41 (0.32–0.50)	0.86 (0.85–0.87)
**Prehospital qSOFA (95% CI)**	≥ 1	0.65 (0.62–0.68)	0.15	0.93 (0.90–0.95)	0.24 (0.22–0.26)	0.18 (0.16–0.20)	0.95 (0.93–0.97)
	≥ 2			0.57 (0.52–0.62)	0.66 (0.64–0.68)	0.23 (0.21–0.26)	0.89 (0.88–0.91)
	≥ 3			0.14 (0.10–0.17)	0.95 (0.94–0.96)	0.33 (0.26–0.41)	0.86 (0.84–0.87)
**Combined qSOFA (95% CI)**	≥ 1	0.68 (0.66–0.71)	0.03	0.97 (0.95–0.99)	0.15 (0.13–0.16)	0.17 (0.16–0.19)	0.96 (0.94–0.99)
	≥ 2			0.87 (0.84–0.90)	0.35 (0.33–0.37)	0.20 (0.18–0.21)	0.91 (0.90–0.93)
	≥ 3			0.67 (0.62–0.72)	0.62 (0.60–0.64)	0.24 (0.21–0.27)	0.91 (0.90–0.93)
	≥ 4			0.46 (0.41–0.51)	0.78 (0.76–0.80)	0.27 (0.24–0.31)	0.89 (0.87–0.90)
	≥ 5			0.16 (0.12–0.19)	0.95 (0.94–0.96)	0.35 (0.28–0.42)	0.86 (0.85–0.88)
	≥ 6			0.08 (0.06–0.11)	0.98 (0.97–0.99)	0.44 (0.32–0.55)	0.86 (0.84–0.87)

Abbreviations: ED, Emergency Department; qSOFA, The quick Sequential Organ Failure Assessment; PPV, Positive Predictive Value; NPV, Negative Predictive Value

*We compared the area under the curve (c-statistics) of each score to ED qSOFA using DeLong’s test.

### Sensitivity analysis

Similar results were obtained for the predictive performance of septic shock and in-hospital mortality as that of sepsis (**Tables 4 and 5**). For instance, the predictive performances for septic shock and in-hospital mortality of prehospital and ED qSOFA were also comparable (e.g., c-statistics of prehospital qSOFA versus ED qSOFA for in-hospital mortality [95%CI], 0.69 [0.65–0.72] vs. 0.69 [0.66–0.73], P-value 0.54), and there were no clinically meaningful differences in the sensitivity of prehospital qSOFA ≥2 and ED qSOFA ≥2 for septic shock and in-hospital mortality (e.g., sensitivity of prehospital qSOFA versus ED qSOFA for septic shock [95%CI], 0.64 [0.56–0.72] vs. 0.68 [0.60–0.76]). Although the predictive abilities of combined qSOFA were equivalent to that of ED qSOFA for estimating the risk of septic shock and in-hospital mortality (e.g., c-statistics of combined qSOFA versus ED qSOFA for septic shock [95%CI], 0.75 [0.71–0.79] vs. 0.74 [0.70–0.78], P-value 0.17), combined qSOFA (cutoff, ≥3) was more sensitive than ED qSOFA (cutoff, ≥2) for septic shock and in-hospital mortality (e.g., sensitivity of combined qSOFA versus ED qSOFA for in-hospital mortality [95%CI], 0.72 [0.66–0.78] vs. 0.59 [0.53–0.66]). The ROC curves representing predictive abilities for septic shock and in-hospital mortality of prehospital qSOFA, ED qSOFA, and combined qSOFA are shown in **Fig 2**. Overall, a cutoff value of 3 for combined qSOFA is appropriate for predicting septic shock and in-hospital mortality as well as sepsis in our primary analysis.

**Table 4:** Predictive abilities of the ED qSOFA, prehospital qSOFA, and combined qSOFA for septic shock.

	Cutoff	c-statistics	P-value*	Sensitivity	Specificity	PPV	NPV
**ED qSOFA (95% CI)**	≥ 1	0.74 (0.70–0.78)	-	0.96 (0.92–0.99)	0.27 (0.25–0.29)	0.07 (0.06–0.08)	0.99 (0.98–1.00)
	≥ 2			0.68 (0.60–0.76)	0.69 (0.68–0.71)	0.12 (0.09–0.14)	0.97 (0.97–0.98)
	≥ 3			0.23 (0.16–0.31)	0.96 (0.96–0.97)	0.27 (0.19–0.35)	0.96 (0.95–0.96)
**Prehospital qSOFA (95% CI)**	≥ 1	0.70 (0.66–0.74)	0.0504	0.98 (0.95–1.00)	0.23 (0.21–0.24)	0.07 (0.06–0.08)	0.99 (0.99–1.00)
	≥ 2			0.64 (0.56–0.72)	0.64 (0.62–0.66)	0.10 (0.08–0.11)	0.97 (0.96–0.98)
	≥ 3			0.25 (0.18–0.32)	0.95 (0.94–0.96)	0.22 (0.15–0.29)	0.96 (0.95–0.96)
**Combined qSOFA (95% CI)**	≥ 1	0.75 (0.71–0.79)	0.17	0.99 (0.98–1.00)	0.14 (0.12–0.15)	0.06 (0.05–0.07)	1.00 (0.99–1.00)
	≥ 2			0.94 (0.90–0.98)	0.33 (0.31–0.35)	0.08 (0.06–0.09)	0.99 (0.98–1.00)
	≥ 3			0.75 (0.68–0.82)	0.59 (0.57–0.61)	0.10 (0.08–0.12)	0.98 (0.97–0.98)
	≥ 4			0.59 (0.51–0.68)	0.76 (0.75–0.78)	0.13 (0.10–0.15)	0.97 (0.96–0.98)
	≥ 5			0.29 (0.21–0.36)	0.94 (0.93–0.95)	0.23 (0.16–0.29)	0.96 (0.95–0.97)
	≥ 6			0.17 (0.10–0.23)	0.98 (0.97–0.98)	0.31 (0.20–0.42)	0.95 (0.94–0.96)

Abbreviations: ED, Emergency Department; qSOFA, The quick Sequential Organ Failure Assessment; PPV, Positive Predictive Value; NPV, Negative Predictive Value

*We compared the area under the curve (c-statistics) of each score to ED qSOFA using DeLong’s test.

**Table 5:** Predictive abilities of the ED qSOFA, prehospital qSOFA, and combined qSOFA for in-hospital mortality.

	Cutoff	c-statistics	P-value*	Sensitivity	Specificity	PPV	NPV
**ED qSOFA (95% CI)**	≥ 1	0.69 (0.66–0.73)	-	0.94 (0.90–0.97)	0.28 (0.26–0.29)	0.11 (0.10–0.13)	0.98 (0.97–0.99)
	≥ 2			0.59 (0.53–0.66)	0.70 (0.68–0.72)	0.16 (0.14–0.19)	0.95 (0.94–0.96)
	≥ 3			0.16 (0.11–0.21)	0.96 (0.96–0.97)	0.31 (0.22–0.39)	0.92 (0.91–0.93)
**Prehospital qSOFA (95% CI)**	≥ 1	0.69 (0.65–0.72)	0.54	0.94 (0.91–0.97)	0.23 (0.21–0.25)	0.11 (0.09–0.12)	0.98 (0.96–0.99)
	≥ 2			0.63 (0.56–0.69)	0.65 (0.63–0.67)	0.15 (0.13–0.18)	0.95 (0.94–0.96)
	≥ 3			0.21 (0.16–0.27)	0.95 (0.94–0.96)	0.31 (0.23–0.38)	0.92 (0.91–0.94)
**Combined qSOFA (95% CI)**	≥ 1	0.72 (0.68–0.75)	<0.01	0.97 (0.94–0.99)	0.14 (0.13–0.15)	0.10 (0.09–0.11)	0.98 (0.96–0.99)
	≥ 2			0.92 (0.88–0.95)	0.34 (0.32–0.36)	0.12 (0.11–0.14)	0.98 (0.97–0.99)
	≥ 3			0.72 (0.66–0.78)	0.60 (0.58–0.62)	0.15 (0.13–0.17)	0.96 (0.95–0.97)
	≥ 4			0.50 (0.44–0.57)	0.77 (0.75–0.78)	0.18 (0.15–0.21)	0.94 (0.93–0.95)
	≥ 5			0.25 (0.19–0.31)	0.95 (0.94–0.96)	0.32 (0.25–0.39)	0.93 (0.92–0.94)
	≥ 6			0.12 (0.07–0.16)	0.98 (0.97–0.99)	0.35 (0.24–0.46)	0.92 (0.91–0.93)

Abbreviations: ED, Emergency Department; qSOFA, The quick Sequential Organ Failure Assessment; PPV, Positive Predictive Value; NPV, Negative Predictive Value

*We compared the area under the curve (c-statistics) of each score to ED qSOFA using DeLong’s test.

## Discussion

In this retrospective study of 2,407 adult patients with suspected infection transported by ambulance to the ED of a tertiary medical center, we found that the sum of prehospital and ED qSOFA ≥3 achieved a higher sensitivity of predicting sepsis based on the Sepsis-3 criteria at the ED compared with prehospital qSOFA ≥2 and ED qSOFA ≥2 alone. By using prehospital qSOFA in combination with ED qSOFA, we were able to identify 44 (12%) out of 369 patients with sepsis who would have been missed by ED qSOFA alone. Additionally, this is the first study to investigate the predictive ability of qSOFA in the prehospital and ED setting for the diagnosis of sepsis based on the Sepsis-3 criteria without using any surrogates (e.g., ICD-9 codes and ICD-10 codes) [26–28].

To enhance the quality of prehospital triage, the use of prehospital qSOFA has been discussed. Previous studies showed that prehospital qSOFA was significantly associated with poor prognosis (e.g., in-hospital mortality, ICU admission, and length of ICU stay) in patients with sepsis [4, 5, 26, 29]. Indeed, the predictive abilities for sepsis of prehospital qSOFA and ED qSOFA were comparable in our study, suggesting the potential utility of prehospital vital signs on triage. On the other hand, the usefulness of qSOFA as a screening tool for patients likely to have sepsis has been controversial, as several studies pointed to the insufficient sensitivity of using ED qSOFA alone (16 to 54%) [7, 12, 30], which is also consistent with our results. Hence, the Surviving Sepsis Campaign 2021 has recommended against using qSOFA as a single screening tool for sepsis [13]. However, given the simplicity of qSOFA and sufficient sensitivity of combined qSOFA, the additional use of prehospital qSOFA to ED qSOFA may be a practical way of screening patients for sepsis on arrival at the ED. Considering that repeated measurements of qSOFA were found to improve predictive validity for sepsis in a previous study [14], our results suggest that prehospital and ED vital signs are particularly suitable for two-point measurements to screen the risk of sepsis, which physicians can easily access at the time of ED triage. Our findings extend prior studies by devising a simple and sensitive screening method—the simultaneous use of prehospital qSOFA and ED qSOFA—for sepsis based on the Sepsis-3 criteria in patients with clinically suspected infection at the ED.

The higher sensitivity of the addition of prehospital qSOFA to ED qSOFA to screen for sepsis compared to ED qSOFA alone could be because the combined qSOFA was able to pick up more patients subsequently diagnosed with sepsis among those with qSOFA <2 on arrival at the ED (e.g., patients with prehospital qSOFA of 2 and ED qSOFA of 1). Given the high morbidity and mortality of sepsis [31], the additional use of prehospital qSOFA, which could easily screen as many as an additional 12% of patients at a higher risk of sepsis, is of great significance. Although ED qSOFA ≥1 was more sensitive for the diagnosis of sepsis than ED qSOFA ≥2 or combined qSOFA ≥3, the ED qSOFA cutoff of 1 is not suitable for clinical use due to its high false positive rate. Therefore, combining prehospital and ED qSOFA may be clinically more beneficial than using ED qSOFA alone.

### Limitations

Our study has several limitations. First, as the relationship between vital signs in the prehospital and ED settings may depend on ambulance transport time, further studies are required to investigate whether our findings could be affected by this. Second, our vital signs data contained missing data (2%-33% of the data depending on the variable), which could be a potential source of bias. However, we believe this problem was minimized by using random forest imputation after the exclusion of patients with mostly missing vital signs [21–23]. Third, because all the data on the ED visits were retrospectively collected, there could have been misclassification. However, all the data used in this study were structured by the NEXT Stage ER system, and most of the data were coded with sensitivity and specificity greater than 90% (e.g., chief complaints, comorbidities, medications, and physician diagnoses) [15]. Lastly, since this study was retrospectively conducted in a single center, our findings may have limited transportability. Larger studies involving multiple facilities with different emergency medical systems (e.g., a hospital located in an urban area surrounded by several hospitals) are warranted to validate our findings.

## Conclusions

We found that using prehospital qSOFA in addition to ED qSOFA could efficiently screen patients with suspected infection at the ED for sepsis. Given the limited patient information available on arrival at the ED, prehospital vital signs may be useful in estimating the risk of sepsis in patients with suspected infection.

## Supporting information

S1 Appendix The modified sepsis clinical surveillance definition. According to the Sepsis-3 criteria, sepsis is defined as life-threatening organ dysfunction due to infection, and can be defined as an increase in the Sequential Organ Failure Assessment (SOFA) score of ≥2-point. Given the difficulty of accurately assessing patients’ conditions based on the Sepsis-3 criteria in retrospective studies, Rhee C et al. developed a new definition for retrospective surveillance of sepsis using electrical medical records. We used the definition of the modified sepsis clinical surveillance as described by Rhee C et al. to identify sepsis in patients who presented to the emergency department with suspected infection.
(DOCX)

## References

[1] Cinel I, Kasapoglu US, Gul F, Dellinger RP. The initial resuscitation of septic shock. J Crit Care. 2020; 57:108–117. doi: 10.1016/j.jcrc.2020.02.004 32135409

[2] Seymour CW, Gesten F, Prescott HC, Friedrich ME, Iwashyna TJ, Phillips GS, et al. Time to Treatment and Mortality during Mandated Emergency Care for Sepsis. N Engl J Med. 2017; 376(23):2235–2244. doi: 10.1056/NEJMoa1703058 28528569 PMC5538258

[3] Seymour CW, Liu VX, Iwashyna TJ, Brunkhorst FM, Rea TD, Scherag A, et al. Assessment of clinical criteria for sepsis: for the third international consensus definitions for sepsis and septic shock (Sepsis‐3). JAMA. 2016; 315(8):762–774. doi: 10.1001/jama.2016.0288 26903335 PMC5433435

[4] Shu E, Ives Tallman C, Frye W, Boyajian JG, Farshidpour L, Young M, et al. Pre-hospital qSOFA as a predictor of sepsis and mortality. Am J Emerg Med. 2019; 37(7):1273–1278. doi: 10.1016/j.ajem.2018.09.025 30322666

[5] Koyama S, Yamaguchi Y, Gibo K, Nakayama I, Ueda S. Use of prehospital qSOFA in predicting in-hospital mortality in patients with suspected infection: A retrospective cohort study. PLoS One. 2019; 14(5):e0216560. doi: 10.1371/journal.pone.0216560 31063494 PMC6504075

[6] Tusgul S, Carron PN, Yersin B, Calandra T, Dami F. Low sensitivity of qSOFA, SIRS criteria and sepsis definition to identify infected patients at risk of complication in the prehospital setting and at the emergency department triage. Scand J Trauma Resusc Emerg Med. 2017; 25(1):108. doi: 10.1186/s13049-017-0449-y 29100549 PMC5670696

[7] Dorsett M, Kroll M, Smith CS, Asaro P, Liang SY, Moy HP. qSOFA has poor sensitivity for prehospital identification of severe sepsis and septic shock. Prehosp Emerg Care. 2017; 21(4):489–498. doi: 10.1080/10903127.2016.1274348 28121217

[8] Brink A, Alsma J, Verdonschot RJCG, Rood PPM, Zietse R, Lingsma HF, et al. Predicting mortality in patients with suspected sepsis at the Emergency Department; A retrospective cohort study comparing qSOFA, SIRS and National Early Warning Score. PLoS One. 2019; 14(1), e0211133. doi: 10.1371/journal.pone.0211133 30682104 PMC6347138

[9] Wang C, Xu R, Zeng Y, Zhao Y, Hu X. A comparison of qSOFA, SIRS and NEWS in predicting the accuracy of mortality in patients with suspected sepsis: A meta-analysis. PLoS One. 2022; 17(4), e0266755. doi: 10.1371/journal.pone.0266755 35427367 PMC9012380

[10] Fernando SM, Tran A, Taljaard M, Cheng W, Rochwerg B, Seely AJE, et al. Prognostic accuracy of the quick sequential organ failure assessment for mortality in patients with suspected infection: a systematic review and meta‐analysis. Ann Intern Med. 2018; 168(4):266–275. doi: 10.7326/M17-2820 29404582

[11] Herwanto V, Shetty A, Nalos M, Chakraborty M, McLean A, Eslick GD, et al. Accuracy of quick sequential organ failure assessment score to predict sepsis mortality in 121 studies including 1,716,017 individuals: a systematic review and meta‐analysis. Crit Care Explor. 2019; 1(9):e0043. doi: 10.1097/CCE.0000000000000043 32166285 PMC7063937

[12] Serafim R, Gomes JA, Salluh J, Póvoa P. A comparison of the Quick‐ SOFA and systemic inflammatory response syndrome criteria for the diagnosis of sepsis and prediction of mortality: a systematic review and meta‐analysis. Chest. 2018; 153(3):646–655. doi: 10.1016/j.chest.2017.12.015 29289687

[13] Evans L, Rhodes A, Alhazzani W, Antonelli M, Coopersmith CM, French C, et al. Surviving sepsis campaign: international guidelines for management of sepsis and septic shock 2021. Intensive Care Med. 2021; 47(11):1181–1247. doi: 10.1007/s00134-021-06506-y 34599691 PMC8486643

[14] Kievlan DR, Zhang LA, Chang CH, Angus DC, Seymour CW. Evaluation of Repeated Quick Sepsis-Related Organ Failure Assessment Measurements Among Patients With Suspected Infection. Crit Care Med. 2018; 46(12):1906–1913. doi: 10.1097/CCM.0000000000003360 30130261 PMC6309444

[15] Goto T, Hara K, Hashimoto K, Soeno S, Shirakawa T, Sonoo T, et al. Validation of chief complaints, medical history, medications, and physician diagnoses structured with an integrated emergency department information system in Japan: the Next Stage ER system. Acute Med Surg. 2020; 7(1):e554. doi: 10.1002/ams2.554 32884825 PMC7453131

[16] Shahsavarinia K, Moharramzadeh P, Arvanagi RJ, Mahmoodpoor A. qSOFA score for prediction of sepsis outcome in emergency department. Pak J Med Sci. 2020; 36(4):668–672. doi: 10.12669/pjms.36.4.2031 32494253 PMC7260919

[17] Rhee C, Kadri S, Huang SS, Murphy MV, Li L, Platt R, et al. Objective Sepsis Surveillance Using Electronic Clinical Data. Infect Control Hosp Epidemiol. 2016; 37(2):163–171. doi: 10.1017/ice.2015.264 26526737 PMC4743875

[18] Delahanty RJ, Alvarez J, Flynn LM, Sherwin RL, Jones SS. Development and Evaluation of a Machine Learning Model for the Early Identification of Patients at Risk for Sepsis. Ann Emerg Med. 2019; 73(4):334–344. doi: 10.1016/j.annemergmed.2018.11.036 30661855

[19] Osawa I, Sonoo T, Soeno S, Hara K, Nakamura K, Goto T. Clinical performance of early warning scoring systems for identifying sepsis among anti-hypertensive agent users. Am J Emerg Med. 2021; 48:120–127. doi: 10.1016/j.ajem.2021.03.091 33878566

[20] Shibata J, Osawa I, Ito H, Soeno S, Hara K, Sonoo T, et al. Risk factors of sepsis among patients with qSOFA<2 in the emergency department. Am J Emerg Med. 2021 Dec;50:699–706. 34879489 10.1016/j.ajem.2021.09.035

[21] missForest: nonparametric missing value imputation using random forest. Available at: https://cran.r-project.org/web/packages/missForest/index.html. [Accessed December 9, 2022]

[22] Shah AD, Bartlett JW, Carpenter J, Nicholas O, Hemingway H. Comparison of random forest and parametric imputation models for imputing missing data using MICE: a CALIBER study. Am J Epidemiol. 2014; 179(6):764–74. doi: 10.1093/aje/kwt312 24589914 PMC3939843

[23] Stekhoven DJ, Bühlmann P. MissForest—non-parametric missing value imputation for mixed-type data. Bioinformatics. 2012; 28(1):112–118. doi: 10.1093/bioinformatics/btr597 22039212

[24] DeLong ER, DeLong DM, Clarke-Pearson DL. Comparing the areas under two or more correlated receiver operating characteristic curves: a nonparametric approach. Biometrics. 1988; 44(3):837–45. 3203132

[25] R Core Team. R: a language and environment for statistical computing. https://www.r-project.org. [Accessed December 9, 2022]

[26] Hirose T, Katayama Y, Ogura H, Umemura Y, Kitamura T, Mizushima Y, et al. Relationship between the prehospital quick Sequential Organ Failure Assessment and prognosis in patients with sepsis or suspected sepsis: a population-based ORION registry. Acute Med Surg. 2021; 8(1):e675. doi: 10.1002/ams2.675 34408882 PMC8360304

[27] April MD, Aguirre J, Tannenbaum LI, Moore T, Pingree A, Thaxton RE, et al. Sepsis Clinical Criteria in Emergency Department Patients Admitted to an Intensive Care Unit: An External Validation Study of Quick Sequential Organ Failure Assessment. J Emerg Med. 2017; 52(5):622–631. doi: 10.1016/j.jemermed.2016.10.012 27823893

[28] Churpek MM, Snyder A, Han X, Sokol S, Pettit N, Howell MD, et al. Quick Sepsis-related Organ Failure Assessment, Systemic Inflammatory Response Syndrome, and Early Warning Scores for Detecting Clinical Deterioration in Infected Patients outside the Intensive Care Unit. Am J Respir Crit Care Med. 2017; 195(7):906–911. doi: 10.1164/rccm.201604-0854OC 27649072 PMC5387705

[29] Barbara P, Graziano C, Caputo W, Litvak I, Battinelli D, Hahn B. The quick sequential organ failure assessment (qSOFA) identifies septic patients in the out-of-hospital setting. Am J Emerg Med. 2018; 36(6):1022–1026. doi: 10.1016/j.ajem.2018.01.073 29426799

[30] Raith EP, Udy AA, Bailey M, McGloughlin S, MacIsaac C, Bellomo R, et al. Prognostic Accuracy of the SOFA Score, SIRS Criteria, and qSOFA Score for In-Hospital Mortality Among Adults With Suspected Infection Admitted to the Intensive Care Unit. JAMA. 2017; 317(3):290–300. doi: 10.1001/jama.2016.20328 28114553

[31] Rudd KE, Johnson SC, Agesa KM, Shackelford KA, Tsoi D, Kievlan DR, et al. Global, regional, and national sepsis incidence and mortality, 1990–2017: analysis for the Global Burden of Disease Study. Lancet. 2020;395(10219):200–211. doi: 10.1016/S0140-6736(19)32989-7 31954465 PMC6970225

