# Supplementary material for: The prognostic utility of prehospital qSOFA in addition to emergency department qSOFA for sepsis in patients with suspected infection: A retrospective cohort study
Source: PLoS One. 2023 Feb 24;18(2):e0282148. doi: 10.1371/journal.pone.0282148 (PMC9956063; doi:10.1371/journal.pone.0282148)
Supplement: S1 Appendix — According to the Sepsis-3 criteria, sepsis is defined as life-threatening organ dysfunction due to infection, and can be defined as an increase in the Sequential Organ Failure Assessment (SOFA) score of ≥2-point. Given the difficulty of accurately assessing patients’ conditions based on the Sepsis-3 criteria in retrospective studies, Rhee C et al. developed a new definition for retrospective surveillance of sepsis using electrical medical records. We used the definition of the modified sepsis clinical surveillance as described by Rhee C et al. to identify sepsis in patients who presented to the emergency department with suspected infection. (DOCX) [file pone.0282148.s001.docx]

**Supplementary information for:**

**The prognostic utility of prehospital qSOFA in addition to emergency department qSOFA for sepsis in patients with suspected infection: A retrospective cohort study**

Ayaka Saito, MD ^1^

Itsuki Osawa, MD ^2^

Junichiro Shibata ^3^

Tomohiro Sonoo, MD ^4,5^

Kensuke Nakamura, MD, PhD ^5^

Tadahiro Goto, MD, MPH, PhD ^4,6^

**Affiliations:**

1. Saku Central Hospital Advanced Care Center, Nagano, Japan
2. Department of Emergency and Critical Care Medicine, The University of Tokyo Hospital, Tokyo, Japan
3. Faculty of Medicine, The University of Tokyo, Tokyo, Japan
4. TXP Medical Co. Ltd., Tokyo, Japan
5. Department of Emergency and Critical Care Medicine, Hitachi General Hospital, Ibaraki, Japan
6. Department of Clinical Epidemiology and Health Economics, School of Public Health, The University of Tokyo, Tokyo, Japan

Supplementary information content

[S1 Appendix. The modified sepsis clinical surveillance definition 3](#_Toc121907211)

# **S1 Appendix. The modified sepsis clinical surveillance definition**

| **1. Presumed serious infection:** |
| --- |
| ・Blood culture obtained (regardless of result), AND  ・New antibiotics administration started within ± 2 days of blood culture day for ≥3 days |
| AND |
| **2. ICU admission:** |
| AND |
| **3. Acute organ dysfunction (any 1 of the following criteria within** ± **2 days of blood culture day):** |
| ・Vasopressor initiation (epinephrine, norepinephrine, phenylephrine, vasopressin, or dopamine)  ・Mechanical ventilation initiation  ・Doubling of serum creatinine level from baseline*  ・Total bilirubin level ≥2.0 mg/dL and doubling from baseline*  ・Platelet count <100 ×10^3^ /μL and ≥50% decrease from baseline (baseline must be ≥100 ×10^3^ /μL)  ・Serum lactate ≥2.0 mmol/L    * Baseline laboratory values refer to the best values during the hospitalization even when the baseline values were taken from previous medical records. |

According to the Sepsis-3 criteria, sepsis is defined as life-threatening organ dysfunction due to infection, and can be defined as an increase in the Sequential Organ Failure Assessment (SOFA) score of ≥2-point. Given the difficulty of accurately assessing patients’ conditions based on the Sepsis-3 criteria in retrospective studies, Rhee C et al. developed a new definition for retrospective surveillance of sepsis using electrical medical records. We used the definition of the modified sepsis clinical surveillance as described by Rhee C et al. to identify sepsis in patients who presented to the emergency department with suspected infection.
